# Surgical and percutaneous revascularization outcomes based on SYNTAX I, II, and residual scores: a long-term follow-up study

**DOI:** 10.1186/s13019-021-01616-6

**Published:** 2021-09-03

**Authors:** Eduardo Bello Martins, Whady Hueb, David L. Brown, Thiago Luis Scudeler, Eduardo Gomes Lima, Paulo Cury Rezende, Paulo Rogério Soares, Cibele Larrosa Garzillo, Jaime Paula Pessoa Linhares Filho, Daniel Valente Batista, Jose Antonio Franchini Ramires, Roberto Kalil Filho

**Affiliations:** 1 Instituto do Coracao (InCor), Hospital das Clinicas HCFMUSP, Faculdade de Medicina, Universidade de Sao Paulo, Sao Paulo, SP Brazil; 2 Washington University School of Medicine, St. Louis, MO USA

**Keywords:** Coronary artery disease, Coronary artery bypass grafting, Coronary angiography, Prognosis

## Abstract

**Background:**

The objective of this study was to evaluate the association of SYNTAX scores I, II, and residual with cardiovascular outcomes of patients undergoing coronary artery bypass grafting (CABG) or percutaneous coronary intervention (PCI) and compare both procedures in a long-term follow-up.

**Methods:**

This is a retrospective single-center study from the MASS registry at the Heart Institute of the University of São Paulo, Brazil in which 969 patients with stable coronary artery disease undergoing CABG (559) or PCI (410) were included. We assessed the SYNTAX scores I, II and residual in both interventions. Clinical endpoints were the first occurrence of a composite of overall death, myocardial infarction, stroke, or repeat revascularization (MACCE) and the total occurrence of each component of MACCE.

**Results:**

In the CABG sample, SSI had a median of 23 (IQR 17–29.5), median SSII of 25.4 (IQR 19.2–32.8), and median rSS of 2 (IQR 0–6.5); in PCI SSI had a median of 14 (IQR 10–19.1), median SSII of 28.7 (IQR 23–34.2), and median rSS of 4.7 (IQR 0–9). Total of 174 events were documented and CABG patients had a lower rate of MACCE (15.6% vs. 21.2%; adjusted HR 1.98; 95% CI 1.13–3.47; *P* = 0.016) and repeat revascularization (3.8% vs. 11.5%; adjusted HR 4.35; CI 95% 1.74–10.85; *P* = 0.002) compared with PCI. No SYNTAX score tertile found a difference in death rate between procedures. In a multivariate analysis, the rSS was an independent predictor for MACCE (HR 1.04; 95% CI 1.01–1.06; *P* = 0.001). Regarding death, the only independent predictors were ejection fraction and renal function.

**Conclusion:**

Surgical revascularization resulted in a more complete revascularization and lower rates of major cardiac or cerebrovascular events in a long-term follow-up. Also, grading the incompleteness of revascularization through the residual SYNTAX score identified a higher event rate, suggesting that complete revascularization is associated with a better prognosis.

**Supplementary Information:**

The online version contains supplementary material available at 10.1186/s13019-021-01616-6.

## Background

Prognostic scores are frequently used in clinical practice to assist in the prediction of adverse cardiovascular events and to improve the management of patients with stable coronary artery disease (CAD). Yet, mortality in patients with stable CAD is similar regardless of the therapeutic strategy, and no score to date has proved useful in the selection of patients to reduce mortality in stable CAD.

Advances in the analysis of the anatomical characteristics of CAD enabled the development of the SYNTAX I score (SSI) [1]. SSI allowed a single tool in assessing the atherosclerotic plaques according to their location, myocardial area at risk, and number and complexity of lesions. Also, its information would help in the choice of the best revascularization procedure as shown in the SYNTAX trial [2].

In populations undergoing percutaneous coronary intervention (PCI), some studies have already found an association of SSI with events, the vast majority of which were in a short- or medium-term follow-up [3–5]. Nevertheless, the prognostic value of SSI in populations undergoing coronary artery bypass grafting (CABG) had inconsistent results in previous studies [4, 6–11].

The lack of clinical variables in the SSI led to the development of SYNTAX II (SSII), which identified independent predictors of mortality from the original population included in the SYNTAX study [12]. The external validation of this score in a large registry of patients with CAD obtained a good calibration [12].

Another limitation of SSI is its inability to analyze revascularization procedures for completeness because the incompleteness of revascularization is associated with a worse prognosis [13]. To solve this issue, the residual SYNTAX score (rSS) was developed. This score better quantifies the completeness of revascularization by removing from the SSI points of properly treated lesions and was found to be an independent predictor of events after PCI [14]. In the surgical population, the long-term impact of rSS is still unknown.

Considering the scarcity of studies that simultaneously analyze the 3 scores in long-term follow-up, and the controversial prognostic value of SYNTAX I and rSS in CABG, this study aimed to assess the association of the 3 SYNTAX scores and outcomes in CABG or PCI and compare both procedures according to these scores.

## Methods

This is a retrospective study based on a database from “The Medicine, Angioplasty or Surgery Study” (MASS) registry. Data were extracted from medical records of patients with stable CAD undergoing CABG or PCI at the Heart Institute of the University of São Paulo.

All groups were treated with medications, such as antiplatelet, anti-hypertensives, lipid-lowering, and antidiabetic drugs required to achieve adequate values of blood pressure and lipid and glycemic levels recommended by guidelines.

All patients had regular clinical follow-up every 6 months and, if necessary, additional visits were scheduled. Subjects in the PCI group received bare-metal stents (BMS) or drug-eluting stents (DES) as available. PCI was performed according to a standard protocol that included administration of aspirin and a thienopyridine agent before the start of the procedure and for a minimum duration of dual antiplatelet therapy of 1 month, up to 12 months, according to the type of stent used. Besides that, complete anatomic revascularization was recommended as the main goal.

For patients undergoing CABG, the use of the left internal thoracic artery (LIMA) as the first-choice conduit for the LAD and, also, complete anatomical revascularization was encouraged. The use of cardiac extracorporeal circulation was defined at the discretion of the surgical team, but the surgical team had experience in both on-pump and off-pump surgery.

The clinical endpoints were the first occurrence of a composite of overall death, myocardial infarction (MI), stroke, or repeat revascularization (MACCE), and the total occurrence of each component of MACCE.

The SSI and SSII calculations were performed according to the official algorithm available at www.syntaxscore.com. The calculations of SSI were performed by 2 interventional cardiologists with the angiography available before the first revascularization procedure of each patient and clinical data obtained from the medical records for the calculation of SSII. Coronary lesions were visually estimated and only those scored had a stenosis ≥ 50% affecting vessels with a diameter ≥ 1.5 mm. To calculate the rSS of the surgical population, the SSI result was analyzed concomitantly with the surgical reports, and the points of the revascularized lesions were subtracted. In the percutaneous population, the calculation of rSS was performed by analyzing the angiography in which the revascularization procedure was performed. Finally, patients were categorized into 3 groups according to the tertiles obtained in each score (1: low, 2: intermediate, and 3: high).

Quantitative variables are presented as median and interquartile range or mean and standard deviation as appropriate. The normality assumption was evaluated using the Kolmogorov–Smirnov test. Categorical variables are presented as percentages and absolute values. Quantitative variables were compared using the Student’s t-test or Wilcoxon rank-sum test and categorical variables by the chi-square test. Intraobserver and interobserver agreement for the SSI were performed for 30 angiographies according to the kappa coefficient. For the correlation analysis between SSI and rSS, the Spearman coefficient was used.

The outcomes in CABG and PCI were presented as Kaplan–Meier curves, and the groups were compared by using the log-rank test. Analyses comparing both procedures according to the SSI, SSII and rSS tertiles were also performed. Cox-proportional hazards models were used to analyze time to event data adjusting for multiple covariates for the endpoints. Factors adjusted in the Cox-proportional hazards models include age, sex, diabetes, ejection fraction, SYNTAX I, SYNTAX II, residual SYNTAX, low-density lipoprotein, peripheral artery disease, chronic obstructive pulmonary disease, left-main disease, 3-vessel disease, and smoking history. The interaction of sex, number of diseased vessels, diabetes, renal function, ejection fraction, and tertiles of SYNTAX scores I, II, and residual was tested using Cox-proportional hazards models with interaction terms.

Receiver operating characteristic (ROC) curves were performed to assess the accuracy of SSI, SSII, and rSS for MACCE and overall death by calculating areas under the curves (AUC).

An exploratory analysis to identify independent predictors of MACCE and death was performed using clinical, angiographic, and laboratory variables. Variables with *P* < 0.2 values in univariate tests were tested by Cox regression with the backward stepwise method, and only those with a *P* < 0.05 variables remained in the final model.

The tests were performed with significance levels of 5%. Statistical analyses were performed using SPSS 21.0 (IBM®) software for Macintosh.

## Results

**Fig. 1:**
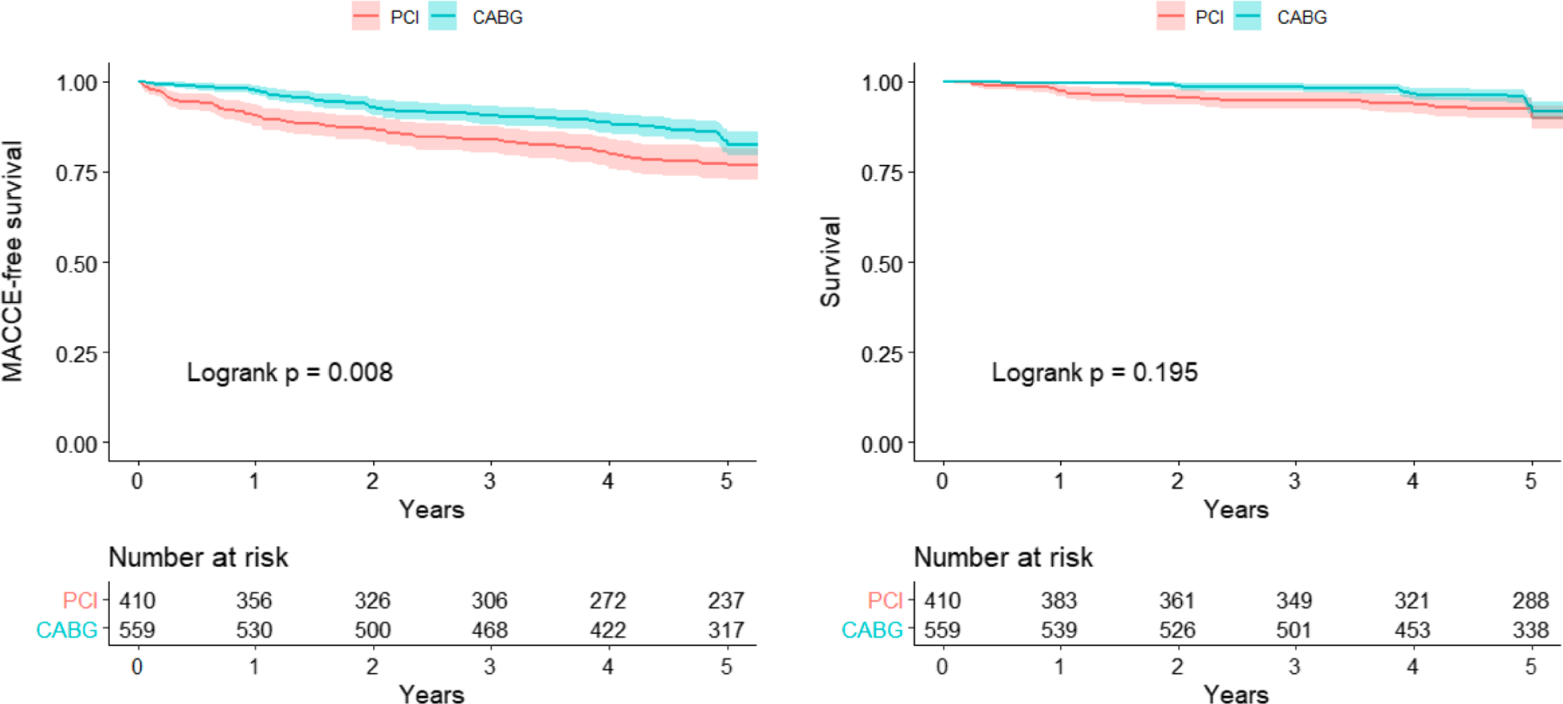
Kaplan–Meier curves showing combined events (MACCE) and death. *CABG* coronary artery bypass grafting, *MACCE* major adverse cardiovascular and cerebrovascular events, *PCI* ercutaneous coronary intervention

In the period from 2008 to 2018, 969 patients underwent a revascularization procedure. Of these, 410 underwent PCI and 559 underwent CABG. The median follow-up of our sample was 5 years (Fig. 1). A cohort flow diagram is presented in the Additional file 1: Figure S1.

In the CABG sample, the SSI median was 23 (17–29.5), SSII median 25.4 (19.2–32.8), and rSS median 2 (0–6.5). A LIMA to LAD was present in 97.7%, and 12.3% received an additional arterial graft, 51.3% of patients were diabetic, and complete revascularization was achieved in 40.1% (Table 1). MACCE occurred in 87 (15.6%), death in 36 (6.4%), MI in 31 (5.5%), repeat revascularization in 21 (3.8%), and stroke in 10 (1.8%) patients (Table 2).

**Table 1:** Baseline characteristics of CABG and PCI samples

Variables	PCI population—410	CABG—559	*P *value
Age (IQR)	61 (54–67)	63 (57–69)	< 0.001
Male sex (%)	258 (62.9)	398 (71.2)	< 0.001
Hypertension (%)	331 (80.9)	444 (79.4)	0.564
Diabetes (%)	247 (60.2)	287 (51.3)	0.006
Smoke history (%)	179 (43.7)	308 (55.1)	< 0.001
EF (IQR)	62.5 (58–69)	60 (51–64)	< 0.001
SYNTAX score I (IQR)	14 (10–19.1)	23 (17–29.5)	< 0.001
Residual SYNTAX score (IQR)	4.7 (0–9)	2 (0–6.5)	< 0.001
SYNTAX score II (IQR)	28.7 (23–34.2)	25.4 (19.2–32.8)	< 0.001
PAD (%)	22 (5.4)	67 (12)	< 0.001
COPD (%)	6 (1.5)	27 (4.8)	0.004
GFR (IQR)	67 (57–80)	69 (58–79)	0.514
LDL (IQR)	117 (89–145)	106 (83–134)	0.002
Aspirin/clopidogrel (%)	406 (99)	553 (98.9%)	0.882
Statin (%)	400 (97.6)	553 (98.9%)	0.099
Three-vessel disease (%)	215 (51)	220 (39.4)	< 0.001
Left-main (%)	15 (3.7)	116 (20.8)	< 0.001
Complete revascularization (%)	106 (25.9)	224 (40.1)	< 0.001
Drug eluting stent (%)	152 (37.1)	N.A	–
On-pump CABG (%)	NA	323 (57.8)	–
Left internal thoracic artery (%)	NA	546 (97.7)	–
Second arterial graft (%)	NA	69 (12.3)	–
Number of grafts (SD)	NA	2.9 (± 0.64)	–

*CABG* coronary artery bypass grafting, *COPD* chronic obstructive pulmonary disease, *EF* ejection fraction, *GFR* glomerular filtration rate, *IQR* interquartile range, *LDL* low density lipoprotein, *NA* non-available, *PAD* peripheral artery disease, *SD* standard deviation

**Table 2:** Event rate in CABG and PCI

Events	CABG (n = 559)	PCI (n = 410)	*P *value
MACCE, n (%)	87 (15.6)	87 (21.2)	0.008
Death, n (%)	36 (6.4)	36 (8.8)	0.195
MI, n (%)	31 (5.5)	28 (6.8)	0.333
Revasc, n (%)	21 (3.8)	47 (11.5)	< 0.001
Stroke, n (%)	10 (1.8)	13 (3.2)	0.174

*MACCE* major adverse cardiac and cerebrovascular events, *MI* myocardial infarction, *Revasc* repeat revascularization

In the PCI group, the SSI median was 14 (10–19.1), SSII median 28.7 (23–34.2), and rSS median 4.7 (0–9). DES was used in 37.1% of patients and 60.2% had diabetes; complete revascularization was achieved in 25.9% (Table 1). MACCE occurred in 87 (21.2%), death in 36 (8.8%), MI in 28 (6.8%), repeat revascularization in 47 (11.5%), and stroke in 13 (3.2%) patients (Table 2).

Before stratification according to SYNTAX scores, a comparison between CABG and PCI whole cohorts found a significant lower difference for MACCE (15.6% vs. 21.2%, *P* log-rank = 0.008; adjusted hazard ratio 1.984; 95% CI 1.134–3.470; *P* = 0.016) and repeat revascularization (3.8% vs. 11.5%, *P* log-rank < 0.001; adjusted hazard ratio 4.356; 95% CI 1.749–10.860; *P* = 0.002; respectively) in the surgical group. The death rate was similar between PCI vs. CABG (8.8% vs. 6.4%, *P* log-rank = 0.195) (Fig. 1; Table 2).

### SYNTAX score I

Analyses of intraobserver (kappa: 0.604, 95% CI 0.269–0.787, *P* < 0.001) and interobserver variability (kappa: 0.660, 95% CI 0.390–0.825, *P* < 0.001) for the SSI were at least moderate. The SSI tertiles obtained were a low SSI ≤ 15 (n = 328), intermediate SSI 15–24 (n = 340), and high SSI > 24 (301). CABG sample had 89, 214, and 256 patients in low, intermediate, and high SSI tertiles, respectively. In PCI, 239, 126, and 45 patients were found in low, intermediate, and high SSI tertiles.

The composite outcome in the lower tertile of SSI was not significantly different between CABG and PCI: 12 (13.5%) versus 43 (18%), *P* = 0.136. The only significant difference found was for the individual analysis of repeat revascularization: n = 3 (3.4%) in CABG versus 27 (11.3%) in PCI (*P* log-rank = 0.021; adjusted hazard ratio, 7.071; CI 95%, 1.367–36.561; *P* = 0.020). No difference was found in the individual analysis of death (*P* = 0.227), myocardial infarction (*P* = 0.637), or stroke (*P* = 0.222) (Additional file 1: Table S1).

In the intermediate SSI tertile, the primary outcome was significantly different between CABG and PCI, with an event rate of 25 (11.7%) in CABG versus 29 in PCI (23%) (*P* log-rank = 0.002; adjusted hazard ratio, 2.704; 95% CI 0.967–7.563; *P* = 0.58). There was a difference for myocardial infarction: n = 8 (3.7%) in CABG versus n = 15 (11.9%) (log-rank, *P* = 0.002; adjusted hazard ratio, 3.131; 95% CI 0.556–17.637; *P* = 0.196). Repeat revascularization rate was also higher in PCI: n = 7 (3.3%) in CABG versus n = 12 (9.5%) in PCI (log-rank, *P* = 0.008; adjusted hazard ratio, 3.061; 95% CI 0.587–15.963; *P* = 0.184). No difference was found in the individual analysis of death (*P* = 0.201) or stroke (*P* = 0.419) (Additional file 1: Table S1).

MACCE rate was significantly different between CABG and PCI in the higher tertile of SSI: n = 50 (19.5%) in CABG versus n = 15 (33.3%) in PCI (*P* log-rank = 0.016; adjusted hazard ratio, 5.157; 95% CI 1.698–15.659; *P* = 0.004). Repeat revascularization was higher in PCI, n = 9 (20%) versus CABG, n = 10 (3.9%) (log rank *P* < 0.001; adjusted hazard ratio, 89.909; 95% CI 11.672–692.570; *P* < 0.001). Stroke rate was also significantly different: n = 6 (2.3%) in CABG and 5 (11.1%) in PCI (log rank *P* = 0.004; adjusted HR 10.132; 95% CI 0.592–179.726; *P* = 0.110). No difference was found in the individual analysis of death (*P* = 0.325) or myocardial infarction (*P* = 0.986) **(**Additional file 1: Table S1).

### SYNTAX score II

The SSII tertiles obtained were a low SSII ≤ 23 (n = 330), intermediate SSII 23-31.3 (n = 320), and high SSII > 31.3 (319). CABG group had 89, 214, and 256 patients in low, intermediate, and high SSII tertiles, respectively. In PCI, 239, 126, and 45 patients were in low, intermediate, and high SSII tertiles.

The composite outcome in the lower tertile of SSII was not significantly different between CABG and PCI: 26 (11.4%) versus 19 (18.6%) (*P* = 0.055). Repeat revascularization was higher in PCI: n = 3 (1.3%) events in CABG versus n = 27 (11.3%) in PCI (*P* log-rank < 0.001; adjusted hazard ratio 5.402; 95% CI 0.472–61.822; *P* = 0.175). No difference was found in the individual analysis of death (*P* = 0.113), MI (*P* = 0.799), and stroke (*P* = 0.644) (Additional file 1: Table S2).

In the intermediate SSII tertile, the primary outcome was not significantly different between CABG and PCI with an event rate of 31 (17.8%) in CABG versus 28 (19.2%) in PCI (*P* = 0.568). No difference was found for death (*P* = 0.295), MI (*P* = 0.329), repeat revascularization (*P* = 0.156), or stroke, n = 4 (*P* = 0.773) (Additional file 1: Table S2).

MACCE rate was statistically similar between CABG and PCI in the higher tertile of SSII: n = 30 (19.1%) in CABG versus n = 40 (24.7%) in PCI (*P* = 0.132). A significant difference was found only for repeat revascularization: n = 7 (4.5%) in CABG versus n = 23 (14.2%) in PCI (*P* log-rank = 0.002; adjusted hazard ratio 12.607; 95% CI 2.848–55.800; *P* = 0.001). There was no difference in the individual analysis of death (*P* = 0.172), myocardial infarction (*P* = 0.972), or stroke (*P* = 0.234) (Additional file 1: Table S2).

### Residual SYNTAX score

A correlation evaluation between SSI and rSS was performed for both surgical procedures. A moderate correlation was found after PCI (R = 0.686, *P* < 0.001), but it was weak after CABG (R = 0.200, *P* < 0.001).

Residual SYNTAX score tertiles were low rSS = 0 (n = 330), intermediate rSS 0–6 (n = 337), and high rSS > 6 (302). CABG group had 224, 195, and 140 patients in low, intermediate, and high SSII tertiles, respectively. In PCI, 106, 142, and 162 patients were in low, intermediate, and high SSII tertiles.

MACCE rate of low rSS tertile was n = 26 (11.6%) in CABG and n = 14 (13.2%) in PCI (*P* = 0.533). No difference was found in the individual analysis of death (*P* = 0.795), MI (*P* = 0.673), repeat revascularization (*P* = 0.275), or stroke (*P* = 0.407) (Additional file 1: Table S3).

Intermediate rSS tertile had a primary outcome rate not significantly different between CABG and PCI: n = 33 (16.9%) in CABG versus n = 35 (24.6%) in PCI (*P* = 0.066). The only difference found was for repeat revascularization: n = 8 (4.1%) in CABG versus n = 22 (15.5%) in PCI (log-rank, *P* < 0.001; adjusted hazard ratio 6.008; 95% CI 1.435–25,152; *P* = 0.014). No difference occurred for death (*P* = 0.269), MI (*P* = 0.663), or stroke (*P* = 0.751) (Additional file 1: Table S3).

In the higher tertile of rSS, the MACCE rate was statistically similar between groups: n = 28 (20%) in CABG versus n = 38 (23.5%) in PCI (*P* = 0.291). A significant difference was found for repeat revascularization: n = 7 (5%) in CABG versus n = 21 (13%) in PCI (*P* log-rank = 0.012; adjusted hazard ratio 12.604; 95% CI 2.524–62.949; *P* = 0.002). No difference occurred in the analysis of death (*P* = 0.497), MI (*P* = 0.621), or stroke (*P* = 0.571) **(**Additional file 1: Table S3).

### SYNTAX scores accuracy, subgroup, and multivariate analysis

**Fig. 2:**
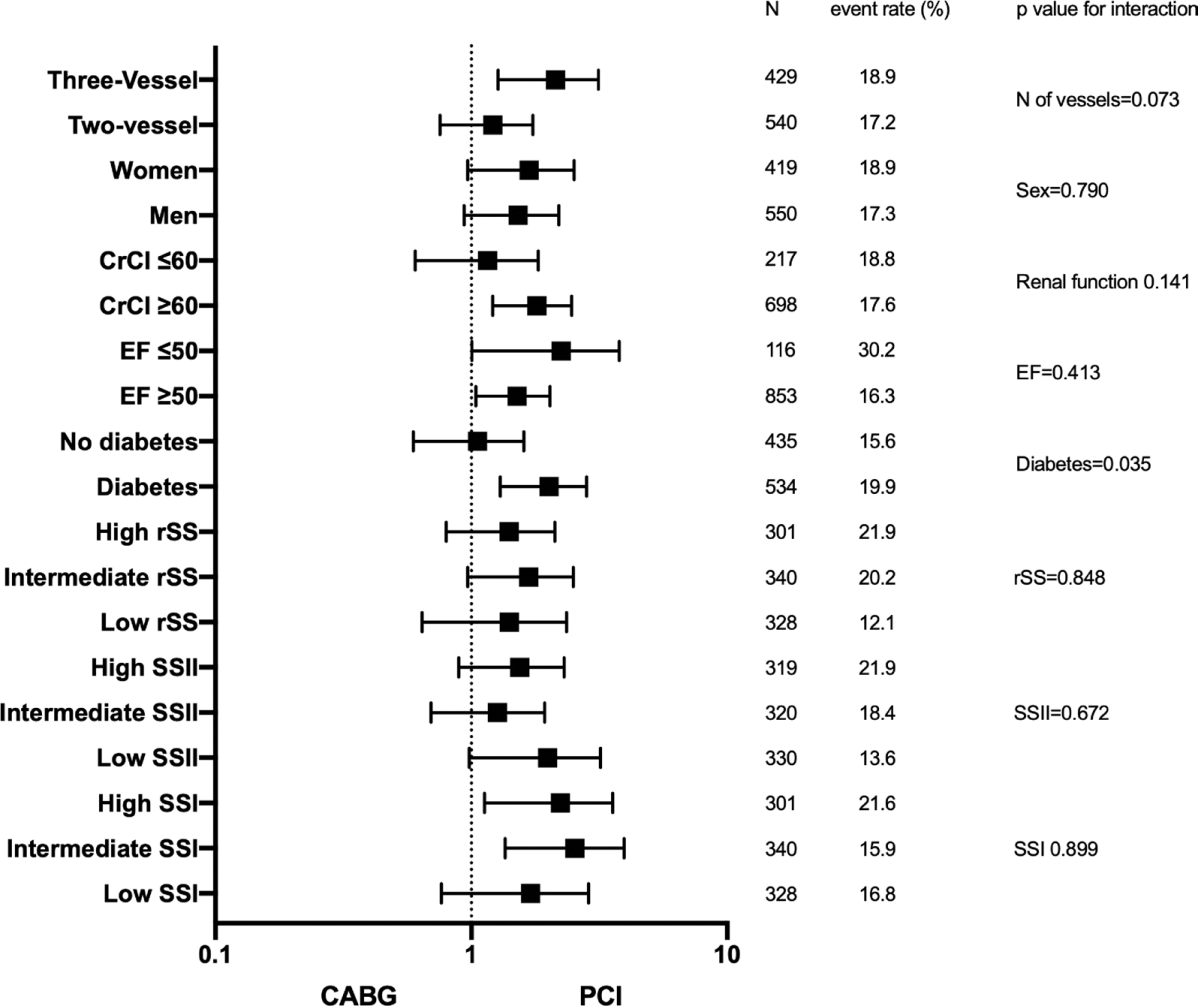
Subgroup analysis for MACCE. *CABG* coronary artery bypass grafting, *CrCl* creatine clearance, *EF* ejection fraction, *MACCE* major adverse cardiovascular and cerebrovascular events, *PCI* percutaneous coronary intervention, *SSI* SYNTAX score I, *SSII* SYNTAX score II, *rSS* residual SYNTAX score

Independent predictors for MACCE in our sample were evaluated in a model testing each component of SSII with rSS, SSI, and other baseline variables. In this sample, the rSS as a continuous variable was an independent predictor for MACCE (hazard ratio 1.042; 95% CI 1.017–1.067; *P* = 0.001) as was EF. For the analysis of death, EF and GFR were the only independent predictors (Table 3). In the subgroup analysis, there was no interaction among the tertiles of the 3 SYNTAX scores and the revascularization procedure, but an interaction for the reduction of MACCE in CABG compared with PCI was found for diabetes (*P* = 0.034) (Fig. 2).

**Table 3:** Univariate and multivariate Cox regression for MACCE and death

Variables	MACCE				Death			
	Univariate analysis		Multivariate analysis					
	HR (CI 95%)	*P* value	HR (95%CI)	*P* value	HR (CI 95%)	*P* value	HR (95%CI)	*P* value
HTN	1.416 (0.939–2.135)	0.097			1.591 (0.816–3.102)	0.173		
DM	1.272 (0.938–1.725)	0.121			1.574 (0.964–2.569)	0.070		
Smoke history	0.861 (0.640–1.160)	0.325			1.061 (0.771–1.460)	0.716		
SSI (each point)^a^	1.014 (0.998–1.030)	0.089			1.010 (0.985–1.036)	0.449		
rSS (each point)^a^	1.043 (1.018–1.067)	0.001	1.042 (1.017–1.067)	0.001	1.013 (0.973–1.055)	0.534		
Age (each point)^a^	1.010 (0.993–1.027)	0.243			1.022 (0.995–1.050)	0.110		
Three-vessel	1.119 (0.831–1.507)	0.460			1.117 (0.703–1.774)	0.640		
PAD	1.079 (0.662–1.757)	0.761			1.527 (0.783–2.978)	0.214		
COPD	0.299 (0.074–1.206)	0.090			0.047 (< 0.001–10.033)	0.264		
GFR (each point)^a^	0.992 (0.983–1.000)	0.060			0.984 (0.971–0.998)	0.025	0.987 (0.973–1.001)	0.029
LDL (each point)^a^	0.998 (0.994–1.001)	0.193			0.998 (0.992–1.003)	0.403		
EF (each point)^a^	0.973 (0.959–0.987)	< 0.001	0.973 (0.960–0.987)	< 0.001	0.972 (0.951–0.993)	0.010	0.972 (0.951–0.993)	0.009
Female sex	1.153 (0.855–1.554)	0.350			0.865 (0.538–1.389)	0.548		
Left-main	1.107 (0.734–1.669)	0.627			1.126 (0.592–2.140)	0.717		

^a^Variables were analyzed continuously. *COPD* chronic obstructive pulmonary disease, *DM* diabetes mellitus, *GFR* glomerular filtration rate, *HTN* hypertension, *LDL* low-density lipoprotein, *MACCE* major cardiovascular and cerebrovascular events, *PAD* peripheral artery disease, *NA* not available

ROC curves were built to examine the accuracy for MACCE and death in our sample**.** SSI AUC was 0.540 (95% CI 0.494–0.586; *P* = 0.095) for MACCE and 0.592 (95% CI 0.448–0.590; *P* = 0.592) for death. SSII AUC was 0.567 (95% CI 0.522–0.613; *P* = 0.005) for MACCE and 0.609 (95% CI 0.541–0.677; *P* = 0.002) for death. RSS AUC was 0.581 (95% CI 0.535–0.627, *P* = 0.001) for MACCE and 0.528 (95% CI 0.462–0.595; *P* = 0.034) for death.

## Discussion

To the best of our knowledge, this is the first study to simultaneously analyze 3 SYNTAX scores in patients undergoing different revascularization procedures. We found a higher rate of MACCE and repeat revascularization in PCI, but there was no statistical difference for mortality between surgical and percutaneous interventions. Furthermore, the application of the three scores found that the incomplete revascularization graded through the rSS was an independent predictor of the composite outcome (MACCE) in both interventions.

MACCE lower rate in CABG compared with that in PCI was mostly driven by a reduction in the repeat revascularization rate, which has been associated in some studies with a worse prognosis [15, 16]. The higher composite event rate in PCI may partially be explained by stent restenosis but also due to a higher level of incomplete revascularization in patients with greater anatomical complexity and atherosclerotic burden. In this regard, we found a higher correlation of SSI and rSS in PCI and no difference for MACCE in the lower tertile of SSI; with the repeat revascularization rate increasing especially in the higher SSI tertile undergoing percutaneous treatment indicating that the SSI has value for event prediction in PCI. Even though SSI was higher in CABG, surgical revascularization was more frequently complete as it is more influenced by the quality of the distal vessel than the anatomic complexity analyzed through SSI.

Incomplete revascularization evaluated by the rSS was a decisive factor for the worse prognosis of the disease. In fact, in a recent meta-analysis, incomplete revascularization was associated with higher rates of events, especially acute myocardial infarction and repeated revascularization [14]. In our data, the incompleteness of myocardial revascularization assessed and graded by the rSS was associated with outcomes even in a population without a high level of residual non-revascularized disease, highlighting the importance of complete revascularization. Even though no interaction occurred between the benefit of CABG and the tertile of rSS, our results suggest that a complete PCI has a similar event rate compared to CABG.

To date, no score has been shown to accurately predict mortality in patients with stable CAD undergoing a revascularization procedure. This study demonstrates that none of the SYNTAX scores were accurately associated with survival, and regardless of the revascularization procedure of choice, there is no difference in death rate. Of note, the SYNTAX trial found a survival difference in favor of CABG in the higher tertile of its sample; however, our sample has a lower disease complexity, which may explain the similar survival rates in the procedures and, in fact, most likely representing the large majority of patients with CAD [3].

Some considerations must be highlighted in understanding the results of the present study. It is a registry-based study with patients from a single quaternary hospital, and patients were referred for surgery or percutaneous treatment; therefore, this sample represents real-world data. Because it was an observational study, we did not have access to the reasons leading to each treatment strategy indication (PCI or CABG). Also, the use of bare metal or DES was at the discretion of the interventionist or the availability of DES, and it could influence the rate of stent restenosis and the repeat revascularization rate.

## Conclusion

Surgical revascularization resulted in a more complete revascularization and lower rates of major cardiac or cerebrovascular events in a long-term follow-up. Also, grading the incompleteness of revascularization through the residual SYNTAX score identified a higher event rate, suggesting that complete revascularization is associated with a better prognosis.

## Supplementary Information

**Additional file 1.Supplementary Figure S1:** Flowchart showing selection of patients included in this study. **Supplementary Table S1:** Event rate in CABG and PCI according to SYNTAX score I. **Supplementary Table S2:** Event rate in CABG and PCI according to SYNTAX score II. **Supplementary Table S3:** Event rate in CABG and PCI according to residual SYNTAX score.

## Data Availability

Please contact the corresponding author for data requests.

## Abbreviations

AUC: Area under the curve
BMS: Bare metal stent
CABG: Coronary artery bypass grafting
COPD: Chronic obstructive pulmonary disease
CrCL: Creatinine clearance
DES: Drug eluting stent
EF: Ejection fraction
GFR: Glomerular filtration rate
HTN: Hypertension
LAD: Left anterior descending
LDL: Low density lipoprotein
LIMA: Left internal mammary artery
PAD: Peripheral artery disease
PCI: Percutaneous coronary intervention
SSI: SYNTAX score I
SSII: SYNTAX score II
ROC: Receiver operating characteristic
rSS: Residual SYNTAX score
