# Supplementary material for: Surgical and percutaneous revascularization outcomes based on SYNTAX I, II, and residual scores: a long-term follow-up study
Source: J Cardiothorac Surg. 2021 Sep 3;16:248. doi: 10.1186/s13019-021-01616-6 (PMC8418036; doi:10.1186/s13019-021-01616-6)
Supplement: Supplementary file 1 — Additional file 1.Supplementary Figure S1: Flowchart showing selection of patients included in this study. Supplementary Table S1: Event rate in CABG and PCI according to SYNTAX score I. Supplementary Table S2: Event rate in CABG and PCI according to SYNTAX score II. Supplementary Table S3: Event rate in CABG and PCI according to residual SYNTAX score. [file 13019_2021_1616_MOESM1_ESM.docx]

Supplementary Figure S1: Flowchart showing selection of patients included in this study.


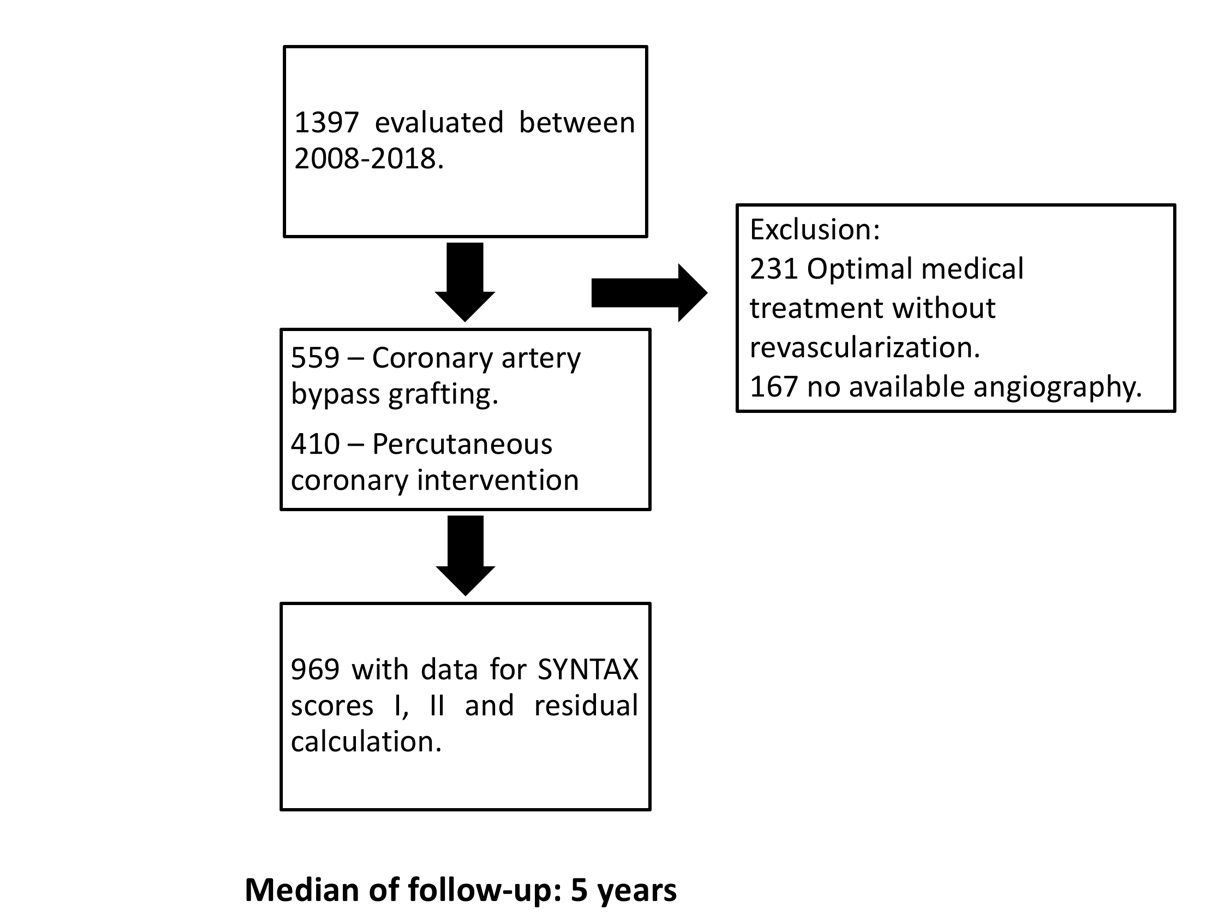


| SYNTAX SCORE II | | | | | | | | | |
| --- | --- | --- | --- | --- | --- | --- | --- | --- | --- |
|  | Low SYNTAX score II (≤23) | | | Intermediate SYNTAX score II (23-31.3) | | | High SYNTAX score II (>31.3) | | |
| Events | CABG (n=228) | PCI (n=102) | p value | CABG (n=174) | PCI (n=146) | p value | CABG (n=157) | PCI (n=162) | p value |
| MACCE - n (%) | 26 (11.4%) | 19 (18.6%) | 0.055 | 31 (17.8%) | 28 (19.2%) | 0.568 | 30 (19.1%) | 40 (24.7%) | 0.132 |
| Death/MI/Stroke – n (%) | 23 (10.1%) | 9 (8.8%) | 0.880 | 22 (12.6%) | 22 (15.1%) | 0.421 | 25 (15.9%) | 29 (17.9%) | 0.415 |
| Death - n (%) | 13 (5.7%) | 2 (2.0%) | 0.113 | 9 (5.2%) | 12 (8.2%) | 0.295 | 14 (8.9%) | 22 (13.6%) | 0.172 |
| MI - n (%) | 4 (1.8%) | 5 (4.9%) | 0.799 | 11 (6.3%) | 13 (8.9%) | 0.329 | 10 (6.4%) | 10 (6.2%) | 0.972 |
| Revasc - n (%) | 3 (1.3%) | 27 (11.3%) | <0.001 | 10 (5.7%) | 14 (9.6%) | 0.156 | 7 (4.5%) | 21 (13%) | 0.004 |
| Stroke - n (%) | 0 (0%) | 2 (2.0%) | 0.664 | 4 (2.3%) | 4 (2.7%) | 0.773 | 3 (1.9%) | 7 (4.3%) | 0.234 |

Supplementary Table S1: Event rate in CABG and PCI according to SYNTAX score I.

P values were calculated with the use of log-rank test. MI, myocardial infarction. MACCE: Major adverse cardiac and cerebrovascular events MI: Myocardial infarction, Revasc: repeat revascularization.

Supplementary Table S2: Event rate in CABG and PCI according to SYNTAX score II.

P values were calculated with the use of log-rank test. MI, myocardial infarction. MACCE: Major adverse cardiac and cerebrovascular events MI: Myocardial infarction, Revasc: repeat revascularization.

| SYNTAX SCORE I | | | | | | | | | |
| --- | --- | --- | --- | --- | --- | --- | --- | --- | --- |
|  | Low SYNTAX score I (≤15) | | | Intermediate SYNTAX score I (15-24) | | | High SYNTAX score I (>24) | | |
| Events | CABG (n=89) | PCI  (239) | p value | CABG (n=214) | PCI (n=126) | p value | CABG (n=256) | PCI (n=45) | p value |
| MACCE- n. / (%) | 12 (13.5%) | 43 (18.0%) | 0.136 | 25 (11.7%) | 29 (23%) | 0.002 | 50 (19.5%) | 15 (33.3%) | 0.016 |
| Death/MI/Stroke – n (%) | 9 (10.1%) | 27 (11.3%) | 0.570 | 20 (9.3%) | 23 (18.3%) | 0.006 | 41 (16%) | 10 (22.3%) | 0.165 |
| Death n./ (%) | 4 (4.5%) | 20 (8.3%) | 0.227 | 10 (4.7%) | 10 (7.9%) | 0.201 | 22 (8.6%) | 6 (13.3%) | 0.325 |
| MI no. / (%) | 5 (5.7%) | 10 (4.2%) | 0.637 | 8 (3.7%) | 15 (11.9%) | 0.002 | 18 (7%) | 3 (6.7%) | 0.986 |
| Revasc. n (%) | 3 (3.4%) | 27 (11.3%) | 0.021 | 7 (3.3%) | 12 (9.5%) | 0.008 | 10 (3.9%) | 9 (20%) | <0.001 |
| Stroke n (%) | 0 (0%) | 4 (1.7%) | 0.222 | 4 (1.9%) | 4 (3.2%) | 0.419 | 6 (2.3) | 5 (11.1%) | 0.004 |

| Residual SYNTAX SCORE | | | | | | | | | |
| --- | --- | --- | --- | --- | --- | --- | --- | --- | --- |
|  | Low residual SYNTAX score (0) | | | Intermediate residual SYNTAX score (0-6) | | | High residual SYNTAX score (>6) | | |
| Events | CABG (n=224) | PCI (n=106) | p value | CABG (n=195) | PCI (n=142) | p value | CABG (n=140) | PCI (n=162) | p value |
| MACCE- n. / (%) | 26 (11.6%) | 14 (13.2%) | 0.533 | 33 (16.9%) | 35 (24.6%) | 0.066 | 28 (20%) | 38 (23.5%) | 0.291 |
| Death/MI/Stroke – n (%) | 22 (9.8%) | 12 (11.3%) | 0.551 | 27 (13.8%) | 23 (16.2%) | 0.449 | 21 (15%) | 25 (15.4%) | 0.656 |
| Death n./ (%) | 13 (5.8%) | 7 (6.6%) | 0.795 | 13 (6.7%) | 15 (10.6%) | 0.269 | 10 (7.1%) | 15 (9.3%) | 0.497 |
| MI no. / (%) | 9 (4.0%) | 5 (4.7%) | 0.673 | 14 (7.2%) | 12 (8.5%) | 0.663 | 8 (5.7%) | 11 (6.8%) | 0.621 |
| Revasc. n (%) | 6 (2.7%) | 5 (4.7%) | 0.275 | 8 (4.1%) | 22 (15.5%) | <0.001 | 7 (5.0%) | 21 (13%) | 0.012 |
| Stroke n (%) | 2 (0.9 | 2 (1.9%) | 0.407 | 3 (1.5%) | 3 (2.1%) | 0.751 | 5 (3.9%) | 8 (4.9%) | 0.571 |

Supplementary Table S3: Event rate in CABG and PCI according to residual SYNTAX score.

P values were calculated with the use of log-rank test. MI, myocardial infarction. MACCE: Major adverse cardiac and cerebrovascular events MI: Myocardial infarction, Revasc: repeat revascularization.
